# INNOVATION CHARACTERISTICS AND STAKEHOLDER INCLUSION IN LTC HOMES DURING COVID-19

**DOI:** 10.1093/geroni/igae098.2161

**Published:** 2024-12-31

**Authors:** Suzanne Santos, Sangduan Ginggeaw, Ruth Melo, Gilciney Andrade Rabello, Franziska Zuniga, Lisa Cranley, Michael Lepore, Charlene Chu

**Affiliations:** University of São Paulo, São Paulo, Sao Paulo, Brazil; University of Massachusetts Amherst, Amhurst, Massachusetts, United States; University of São Paulo, São Paulo, Sao Paulo, Brazil; University of São Paulo, São Paulo, Sao Paulo, Brazil; University of Basel, Basel, Basel-Stadt, Switzerland; University of Toronto, Toronto, Ontario, Canada; University of Massachusetts Amherst, Amherst, Massachusetts, United States; University of Toronto, Toronto, Ontario, Canada

## Abstract

The toll the COVID-19 pandemic has taken on the quality of life of those living and working in Long-Term Care (LTC) homes resulted in calls for innovation in the LTC sector. Results from the observational, experimental, qualitative and mixed-method studies (n=62) showed that 50% and 45.2% of the innovations were characterized as products and processes, respectively. Organizational innovations were reported in 4.8% of the studies. Regarding the settings, the majority (52.4%) of the studies were performed at the micro level, followed by meso (25.4%) and macro (20.6%) levels. The identified innovations were predominantly (35.5%) related to new tools for clinical care and staff education, such as online pain assessment training and the creation of virtual instruments for shared end-of-life care decisions, followed by telecommunications interventions (22.6%), generally aimed at improving connections between residents and their families. COVID-19 detection/prevention (11.3%) was the third most common innovation category in this scoping review. Participants of the studies included staff (71%), residents (56%), family members/caregivers (29%) and experts/researchers (11.3%). Only 9.7% of them included all stakeholders. The innovations implied active engagement (62.9%), where the interventions relied on stakeholder’s actions (e.g. video calls); passive engagement (22.6%), where no actions from stakeholders were needed (e.g. ambient monitoring systems) and co-designed interventions (14.5%), where participants contributed with all stages (planning, implementing, evaluating) of the innovation. Innovations in LTC homes during COVID-19 were mainly characterized as new products or processes aimed at the care of residents and staff training, which required the active involvement of stakeholders, mostly staff.

